# Admissions of Children and Adolescents With Deliberate Self-harm to Intensive Care During the SARS-CoV-2 Outbreak in Australia

**DOI:** 10.1001/jamanetworkopen.2022.11692

**Published:** 2022-05-11

**Authors:** Claire Corrigan, Graeme Duke, Johnny Millar, Eldho Paul, Warwick Butt, Michael Gordon, Jacinta Coleman, David Pilcher, Felix Oberender

**Affiliations:** 1 Paediatric Intensive Care Unit, Monash Children’s Hospital, Melbourne, Australia; 2 Eastern Health Intensive Care Research Centre, Eastern Health, Melbourne, Australia; 3 Eastern Health Clinical School, Monash University, Melbourne, Australia; 4 Paediatric Intensive Care Unit, Royal Children’s Hospital, Melbourne, Australia; 5 Department of Paediatrics, University of Melbourne, Australia; 6 Murdoch Children's Research Institute, University of Melbourne, Melbourne, Australia; 7 Centre for Outcome and Resource Evaluation, Australian and New Zealand Intensive Care Society, Melbourne, Australia; 8 Monash Centre for Health Research and Implementation, School of Public Health and Preventive Medicine, Monash University, Melbourne, Australia; 9 Australian and New Zealand Intensive Care Research Centre, Department of Epidemiology and Preventive Medicine, Monash University, Melbourne; 10 Early in Life Mental Health Service, Monash Health, Melbourne, Australia; 11 Department of Psychiatry, Monash University Melbourne; 12 Department of Adolescent Medicine, Monash Children’s Hospital, Melbourne, Australia; 13 Department of Intensive Care, Alfred Health, Melbourne, Australia; 14 Department of Paediatrics, Monash University, Melbourne, Australia

## Abstract

**Importance:**

Identification of potential indirect outcomes associated with the COVID-19 pandemic in the pediatric population may be essential for understanding the challenges of the current global public health crisis for children and adolescents.

**Objective:**

To investigate whether the SARS-CoV-2 outbreak and subsequent effective public health measures in Australia were associated with an increase in admissions to intensive care units (ICUs) of children and adolescents with deliberate self-harm (DSH).

**Design, Setting, and Participants:**

This national, multicenter cohort study was conducted using the Australian data subset of the binational Australian and New Zealand Paediatric Intensive Care registry, a collaborative containing more than 200 000 medical records with continuous contributions from all 8 Australian specialist, university-affiliated pediatric ICUs, along with 1 combined neonatal-pediatric ICU and 14 general (adult) ICUs in Australia. The study period encompassed 6.5 years from January 1, 2015, to June 30, 2021. Patients aged 12 to 17 years were included. Data were analyzed from December 2021 through February 2022.

**Exposures:**

Any of the following admission diagnoses: ingestion of a drug, ingestion of a nondrug, hanging or strangulation, or self-injury.

**Main Outcomes and Measures:**

The primary outcome measure was the temporal trend for national incidence of DSH ICU admissions per 1 million children and adolescents aged 12 to 17 years in Australia.

**Results:**

A total of 813 children and adolescents aged 12 to 17 years admitted to ICUs with DSH were identified among 64 145 patients aged 0 to 17 years in the Australian subset of the registry during the study period. Median (IQR) age was 15.1 (14.3-15.8) years; there were 550 (67.7%) female patients, 261 (32.2%) male patients, and 2 (0.2%) patients with indeterminate sex. At the onset of the pandemic, monthly incidence of DSH ICU admissions per million children and adolescents increased from 7.2 admissions in March 2020 to a peak of 11.4 admissions by August 2020, constituting a significant break in the temporal trend (odds ratio of DSH ICU admissions on or after vs before March 2020, 4.84; 95% CI, 1.09 to 21.53; *P* = .04). This occurred while the rate of all-cause admissions to pediatric ICUs of children and adolescents of all ages (ie, ages 0-17 years) per 1 million children and adolescents decreased from a long-term monthly median (IQR) of 150.9 (138.1-159.8) admissions to 91.7 admissions in April 2020.

**Conclusions and Relevance:**

This cohort study found that the coronavirus pandemic in Australia was associated with a significant increase in admissions of children and adolescents to intensive care with DSH.

## Key Points

**Question:**

Was the SARS-CoV-2 outbreak in Australia associated with an increase in intensive care unit (ICU) admissions of children and adolescents with severe deliberate self-harm?

**Findings:**

This cohort study identified 813 patients aged 12 to 17 years admitted to pediatric ICUs with deliberate self-harm over 6.5 years. Monthly admissions per million children and adolescents increased significantly at the onset of the pandemic, from 7.2 admissions in March 2020 to 11.4 admissions by August 2020.

**Meaning:**

This study found that the coronavirus pandemic in Australia was associated with a significant increase in admissions of children and adolescents to intensive care with deliberate self-harm.

## Teaser

This cohort study investigates the rate of intensive care admissions with deliberate self-harm among children and adolescents in Australia during the SARS-CoV-2 outbreak.

## Introduction

Suicide is the leading cause of death in Australian children aged between 5 and 17 years (2.5 deaths per 100 000 members of an age-matched Australian population).^1^ More than three-quarters of these suicides occur between ages 15 and 17 years.

Self-injurious thoughts and behaviors include those without actual suicidal intent, as well as suicide.^2,3^ These broad strands can be further differentiated based on underlying thought, implementation, and outcome.^3^ Suicidal ideation and deliberate self-harm (DSH; ie, self-poisoning or self-injury) are common in adolescence.^4,5^ The most common hospital presentations after suicide attempts involve drug ingestion,^6^ while hanging is the most common method of suicide attempts resulting in death.^7^ Suicidal conduct has been associated with behavioral and psychiatric disorders, such as attention-deficit/hyperactivity disorder, anxiety disorders, and depressive disorders.^8,9^ DSH is associated with illegal drug use and alcohol consumption,^4,10^ while suicide among children and adolescents is associated with emotional neglect, emotional abuse, and sexual abuse.^11^ There is also an association between suicide in children and adolescents and mental health disorders or self-harming in other family members.^12^

Social isolation is frequently cited as a factor associated with triggering of self-harming,^13^ particularly when associated with bullying.^4,10,13^ Similarly, traumatic events are known to be factors associated with triggering of pediatric DSH and suicide.^11^ In this context, the COVID-19 pandemic and subsequent public health measures were associated with extraordinary pressures and restrictions on a population level, including for children and adolescents.

The outbreak of novel coronavirus SARS-CoV-2 was declared a pandemic by the World Health Organization on March 11, 2020.^14^ The Australian Federal Government announced a national state of emergency with restrictions on social activity on March 23, 2020.^15^ Stay-at-home orders were implemented by all Australian states and territories. Schools and playgrounds were closed, and social gatherings were prohibited.

These strong and effective public health measures aimed at containing the outbreak carried potential risk for psychological harm, particularly at the extremes of age among older individuals, children, and adolescents.^16,17^ School closures added to family stress, especially when parents were working from home or facing unemployment, loss of income, or disruption to business.^18,19^ Inability to associate with peers can be associated with worsened feelings of isolation and mental health-related behaviors among adolescents.^16^ Domestic violence increased during the coronavirus pandemic.^20^ Online mental health services in Australia reported a 50% increase in young people seeking support during the pandemic compared with the same time in the preceding year.^21^

We investigated changes in admissions to intensive care units (ICUs) of children and adolescents with DSH during the first 15 months of the COVID-19 pandemic in Australia, between March 2020 and June 2021. We aimed to investigate whether the onset of the pandemic was associated with a break from long-term trends in the rate of DSH admissions to pediatric ICUs.

## Methods

This cohort study was approved by the Monash Health Human Research Ethics Committee in accordance with the Australian National Statement on Ethical Conduct in Human Research.^22^ Deidentified data are held by the registry under Part VC (Quality Assurance Confidentiality) of the Health Insurance Act 1973, Commonwealth of Australia.^23^ The Clinical Advisory Committee of the Australian and New Zealand Pediatric Intensive Care (ANZPIC) registry authorized data extraction in accordance with the Australian and New Zealand Intensive Care Society (ANZICS) Centre for Outcome and Resource Evaluation data access and publication policy^24^ and in consultation with the ANZICS Pediatric Study Group. Reporting of study design and results follows the Strengthening the Reporting of Observational Studies in Epidemiology (STROBE) reporting guideline for observational studies.

### Study Setting and Design

The ANZPIC registry is a binational collaborative with continuous contributions from all 8 Australian specialist, university-affiliated pediatric ICUs, along with 1 combined neonatal-pediatric ICU, and 14 general (adult) ICUs in Australia. The data set contains more 200 000 medical records, approximately 94% of all pediatric ICU admissions in Australia and New Zealand.^25^ Data are collected prospectively by participating units and submitted quarterly to the registry.

Using the Australian data subset, we conducted a national, multicenter retrospective cohort study to assess incidence of admission after DSH. Records were included in the study for all patients aged 12 to 17 years who were admitted to an ICU with 1 or more of the following admission diagnoses: ingestion of a drug, ingestion of a nondrug, hanging or strangulation, or self-injury during the 6.5 years from January 1, 2015, to June 30, 2021. DSH was subsequently used as an encompassing term^26^ for all inclusion diagnoses. It did not denote presence or absence of suicidal intent or provide information for subclassification within categories of suicidal and nonsuicidal self-injury. Patients aged younger than 12 years were excluded because DSH and suicide are rare in young children.^3,27^

Patient variables included all admission diagnoses, demographic data, and patient outcomes, including ICU and hospital survival, ICU and hospital length of stay (LOS), and presence or absence of organ-support therapies (ie, mechanical ventilation, vasopressor or inotrope requirement, and kidney-replacement therapy). Risk of death in ICU was estimated using the Pediatric Index of Mortality 3 (PIM3).^28^ Age- and sex-specific monthly population estimates were derived from the yearly data set of the Australian Bureau of Statistics.^29^ Race and ethnicity data, except for indigenous status, are not collected in the ANZPIC registry and were not included in the analysis.

The primary outcome measure was the temporal trend for national incidence of DSH ICU admissions per 1 million children and adolescents aged 12 to 17 years. Secondary outcomes included temporal trends in ICU and hospital LOS, use of organ-support therapies, ICU mortality, and hospital mortality.

### Statistical Analysis

Data were grouped by calendar month of admission. Group data are reported as median (IQR) for nonparametric data, while numbers and percentages are reported for categorical outcomes. Incident rates were calculated for each calendar month as event count per age-matched population.

In the next phase, we undertook time-series analyses to address the presence of significant changes in temporal trends for primary and secondary outcomes. Analyses were performed using Stata statistical software version 17.0 (StataCorp), including the user-specific commands itsa and xtbreak.^30,31^ These commands include adjustment for autocorrelation within complex time-series data, identify temporal trends, and estimate trends within complex cross-sectional time-series data. We used xtbreak to investigate the presence and timing of significant changes in the risk-adjusted incident trend of interest (eMethods 1 in Supplement 1). The presence of a hypothesized trend break in March 2020, at the onset of the COVID-19 pandemic, was also tested. The itsa command quantifies statistical significance and graphs selected break points (eMethods 1 in Supplement 1). It performs an interrupted time-series analysis for group comparisons, observing an outcome variable over multiple, equally spaced time periods before and after the selected intervention time.^30^

Finally, we fitted multivariate regression models to each outcome of interest to test the presence of risk-adjusted linear temporal trends (adjusted for factors including patient severity of illness quantified using PIM3 score, age, sex, and state of origin). Each model was repeated with the inclusion of a binary time covariate (ie, before vs after March 2020) to assess the presence or absence of a pandemic association by comparing Akaike and Bayes information criteria^32^ to investigate which model had the best fit. Mixed effect regression (melogit) was applied to binary outcome variables (ie, DSH admission, death, and organ-support interventions), and negative binomial regression was fitted to continuous outcome variables (ie, PIM3 score and hospital LOS) (eMethods 2 in Supplement 1). *P* values were 2-sided, and a *P* value < .05 was accepted as significant. Data were analyzed from December 2021 through February 2022.

## Results

### Patient Cohorts

The population of children and adolescents aged 12 to 17 years in Australia increased over the study period, from 1.70 million individuals in January 2015 to 1.86 million individuals in June 2021. There were 64 145 admissions of patients aged 0 to 17 years to the ANZPIC registry from Australian ICUs between January 1, 2015, and June 30, 2021. Of these admissions, there were 11 277 children and adolescents aged 12 to 17 years (17.6%), and of these patients, 813 individuals (7.2%) met inclusion criteria (median [IQR] age, 15.1 [14.3-15.8] years; 550 [67.7%] female patients, 261 [32.2%] male patients, and 2 [0.2%] patients with indeterminate sex). There were 42 deaths among patients admitted with DSH (5.2%), compared with 348 deaths among patients admitted from all causes (3.1%). Discharge date was missing from 1 patient, who was excluded from analysis of LOS. There were no other missing data, and all identified patients were included in the primary analysis. Patient characteristics are summarized in Table 1.

**Table 1.** Characteristics of Patients Admitted to Pediatric ICU With Deliberate Self-harm

Characteristic	Patients, No. (%)		
	Whole time series (N = 83)^a^	Prepandemic (n = 583)^b^	Pandemic (n = 230)^c^
Age, median (IQR), y	15.1 (14.3-15.8)	15.0 (14.2-15.7)	15.2 (14.4-15.9)
Sex			
Male	261 (32.2)	186 (31.9)	75 (32.6)
Indeterminate	2 (0.2)	0 (0)	2 (0.9)
Female	550 (67.7)	397 (68.1)	153 (66.5)
LOS, median (IQR)			
ICU, h	26.6 (15.7-44.4)	26.5 (15.8-45.0)	26.9 (15.0-43.4)
Hospital, d	2.6 (1.5-5.9)	2.6 (1.5-5.9)	2.8 (1.6-5.8)
Treatment use			
Mechanical ventilation	400 (49.2)	299 (51.3)	101 (43.9)
Vasopressors or inotropes	107 (13.2)	79 (13.6)	28 (12.2)
Kidney replacement therapy	9 (1.1)	6 (1)	3 (1.3)
Death			
In ICU	40 (4.9)	31 (5.3)	9 (3.9)
In hospital	42 (5.2)	33 (5.7)	9 (3.9)
PIM3 risk of death score, median (IQR)	0.016 (0.012-0.038)	0.031 (0.012-0.040)	0.014 (0.012-0.035)

Abbreviations: ICU, intensive care unit; LOS, length of stay; PIM3, Pediatric Index of Mortality 3.

^a^ January 1, 2015, to June 30, 2021.

^b^ January 1, 2015, to March 30, 2020.

^c^ April 1, 2020, to June 30, 2021.

Of 813 study patients, 230 individuals (15.2%) were admitted during the first 15 months of the COVID-19 pandemic in Australia, between April 1 and June 30, 2021. Ingestion of a drug was the predominant diagnosis (657 patients [80.8%]), followed by hanging or strangulation (80 patients [9.8%]) and nondrug ingestion (40 patients [4.9%]) (Table 2). Other self-injuries (36 patients [4.4%]) included admission diagnoses of isolated trauma, multiple traumas, and burns.

**Table 2.** Crude ICU and Hospital Mortality

Diagnosis	No. (%)^a^		
	Patients	Outcome, death	
		ICU	Hospital
Ingestion of a drug	657 (80.8)	5 (0.8)	7 (1.1)
Hanging or strangulation	80 (9.8)	34 (42.5)	34 (42.5)
Ingestion of a non-drug	40 (4.9)	0	0
Self-injury, other	36 (4.4)	1 (2.8)	1 (2.8)
DSH, total	813 (100)	40 (4.9)	42 (5.2)
ANZPIC registry, all causes^b^	11 277	260 (2.3)	348 (3.1)

Abbreviations: ANZPIC, Australian and New Zealand Paediatric Intensive Care; DSH, deliberate self-harm; ICU, intensive care unit.

^a^ Outcomes are among patients aged 12 to 17 years admitted to pediatric intensive care with DSH between January 1, 2015, and June 30, 2021.

^b^ Registry comparator was ANZPIC registry crude all-cause mortality in patients ages 12 to 17 years.

### Primary Outcome

**Figure 1.**
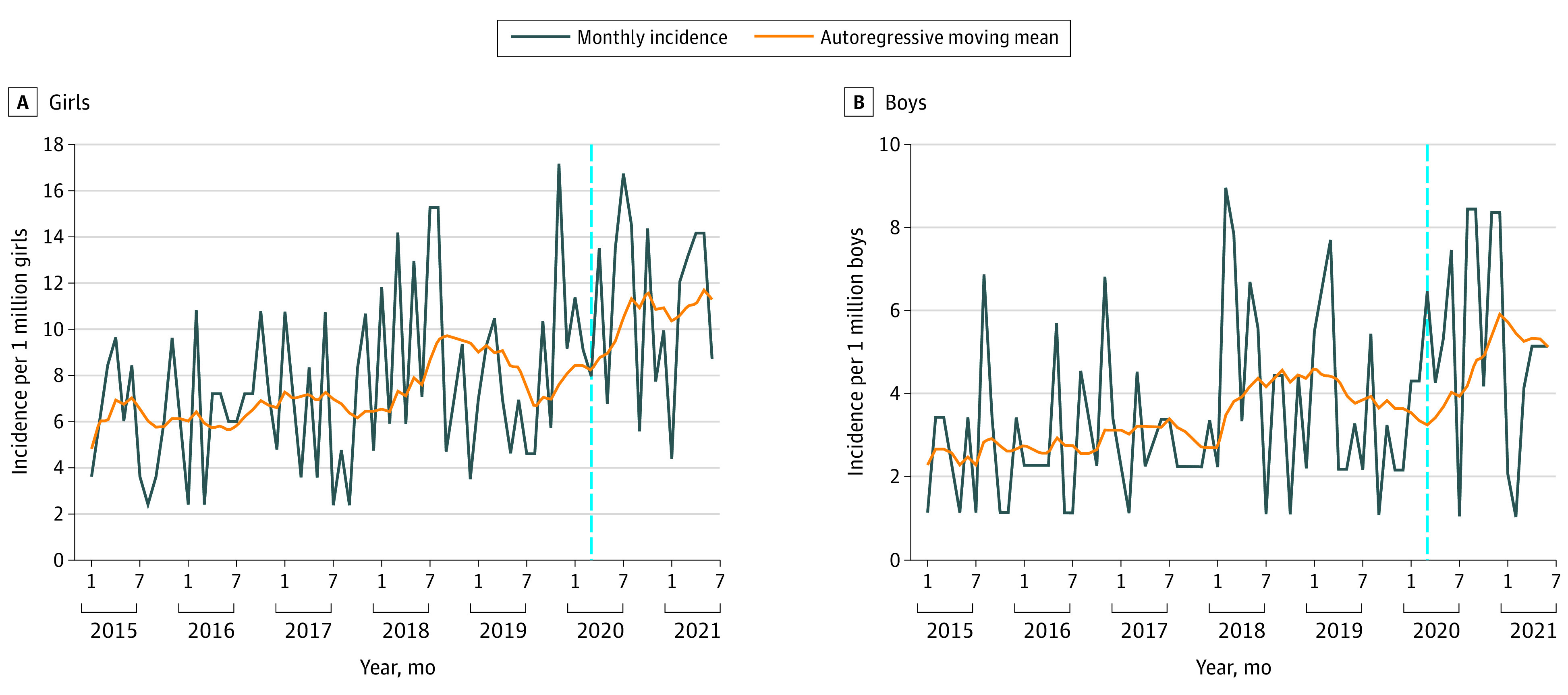
Temporal Trends in DSH ICU Admissions in Boys and Girls DSH indicates deliberate self-harm; ICU, intensive care unit; vertical lines, hypothesized break point in March 2020.

**Figure 2.**
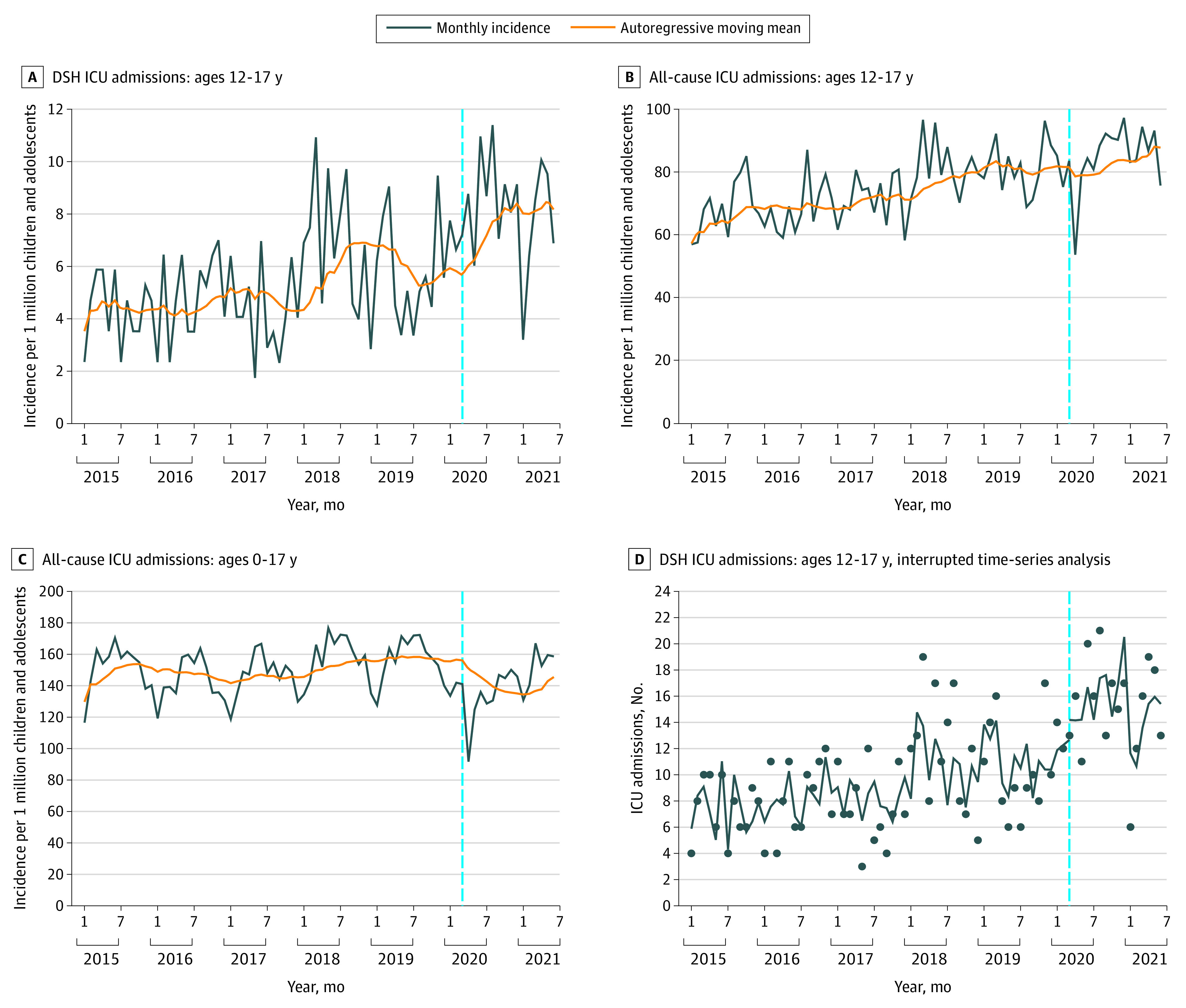
Temporal Trends and Interrupted Time-Series Analysis of ICU Admissions Vertical lines indicate hypothesized break point in March 2020. A, Pediatric intensive care unit (ICU) admission rate with deliberate self-harm (DSH) of children and adolescents aged 12 to 17 years. B, All-cause pediatric ICU admission rate of children and adolescents aged 12 to 17 years. C, All-cause pediatric ICU admission rate of children of all ages (0-17 years). D, Interrupted time-series analysis of DSH ICU admissions of children and adolescents aged 12 to 17 years. Dots indicate observed number of admissions; line, estimated number of admissions.

Temporal trends in incidence of DSH ICU admissions of boys and girls aged 12 to 17 years are depicted in Figure 1. Incidence for girls exceeded that for boys across the study period. Total incidence of ICU admission rates with DSH revealed considerable volatility with patterns of seasonal and nonseasonal changes (Figure 2A). Similar patterns were seen with all-cause ICU admission incidence for the same age group until March 2020 (Figure 2B). This differed from the seasonal pattern apparent over the same period for all-cause and all-age admissions to pediatric ICUs (Figure 2C).

After the hypothesized break point in the timeline of March 2020, coinciding with the onset of the COVID-19 pandemic in Australia, monthly all-cause ICU admission rates for children and adolescents aged 0 to 17 years and 12 to 17 years per million children and adolescents decreased to 91.7 admissions and 53.7 admissions, respectively, in April 2020. These were below the long-term monthly median (IQR) of 150.9 (138.1-159.8) admissions and 78.1 (68.9-84.4) admissions, respectively.

The rate of all-cause ICU admissions of children and adolescents of all ages subsequently remained below prepandemic levels throughout 2020. By contrast, the incidence of ICU admission with DSH per million children and adolescents aged 12 to 17 years increased after this time, from 7.2 admissions in March 2020 to a peak of 11.4 admissions in August 2020. In interrupted time-series and regression analysis (Figure 2D), there was a significant increase in the odds of DSH ICU admissions on or after March 2020 (odds ratio vs before March 2020, 4.84; 95% CI, 1.09 to 21.53; *P* = .04) (eResults in Supplement 1). Time-series analysis using xtbreak, however, also identified that an increase in incidence of DSH admissions may have commenced earlier, at an estimate of October 2019 (95% CI, July 2019 to January 2020).

### Secondary Outcomes

In time-series analysis and linear regression analysis, there was no significant change in long-term trends for secondary outcomes. We were unable to identify interruptions during 2019 or 2020 for any secondary patient outcome. ICU LOS was short, with a median (IQR) of 26.6 (15.7-44.4) hours, as was hospital LOS, with a median (IQR) of 2.6 (1.5-5.9) days (Table 1). Nearly one-half of children and adolescents aged 12 to 17 years admitted to pediatric intensive care with DSH required mechanical ventilation (400 patients [49.2%]). ICU mortality was 4.9% (95% CI, 3.5%-6.7%), and hospital mortality was 5.2% (95% CI, 3.7%-6.9%). Most deaths were from hanging or strangulation (Table 2).

## Discussion

In this cohort study, we undertook a retrospective analysis of temporal trends in admissions to ICU after DSH among pediatric patients aged 12 to 17 years in Australia from January 1, 2015, to June 30, 2021. We identified an increase in the total pediatric population aged 0 to 17 years. After a break point in the timeline in March 2020, coinciding with the onset of the COVID-19 pandemic in Australia and introduction of nationwide public health measures, there was a substantial decrease in all-cause admissions to pediatric ICUs, while ICU admissions of patients aged 12 to 17 years with DSH increased significantly. The DSH ICU admission rate remained increased for the remainder of the pandemic months included in our analysis, except for a sharp decline that coincided with the annual school summer holidays in January, similar to preceding years. This increase in ICU admission rate with DSH among children and adolescents occurred among a patient cohort with particularly concerning characteristics. While LOS in intensive care and in the hospital for children and adolescents identified in our analysis was short, the need for invasive ventilation of 49.2% of these patients contrasts with a mean of 36.1% for all children and adolescents in Australian ICUs.^25^ Similarly, the crude hospital mortality rate of 5.2% for children admitted to intensive care after DSH was notably higher than the mean of 3.1% for all patients aged 12 to 17 years in the registry during the study period.

The increase in DSH requiring admission to intensive care seen in our data substantiates concerns about child and adolescent mental health during the COVID-19 pandemic.^33^ In April 2020, in a survey among adolescents in Wuhan, China, Wie et al^34^ reported increased levels of anxiety and depression associated with stay-at-home restrictions during the city’s COVID-19 outbreak. This was consistent with similar findings among undergraduate students in Beijing and adolescents in Taiwan during the 2003 severe acute respiratory syndrome (SARS) outbreak.^35,36^ Gan et al^37^ noted that coping with stress associated with the infectious disease emergency at that time was characterized by an absence of a good strategy-situation fit. In contrast to other stresses of daily life, epidemic-related stress was predominately perceived as uncontrollable and led to a predominance of emotion-focused coping strategies over problem-focused ones.

The association of the pandemic with an increase in the most severe outcomes of psychopathology as shown in our study is greatly concerning, although not necessarily unexpected. Previous population-level crises, such as the 2008 to 2010 global recession, have been associated with an increase in suicides,^38,39^ although data specific to children and adolescents are lacking. Likewise, infectious disease–related public health emergencies have been associated with an increase in suicidal behavior in adults.^40^ In children and adolescents, isolation and quarantine during the 2009 influenza A (H1N1) pandemic have been associated with a substantially increased risk and rate of posttraumatic stress disorder.^41^ However, mental health outcomes associated with a society-wide crisis are complex. The simple association between economic recession and suicide rates, for example, has been challenged by the Stankunas et al^42^ analysis of trends in postcommunist Baltic states, suggesting a more nuanced interplay of societal factors.^42^ Lau et al^43^ described positive changes in mental health reported by residents of Hong Kong at the end of the 2003 SARS outbreak.

This notwithstanding, our data show a substantial increase in severe outcomes of acute mental health pathology at onset of the pandemic in Australia. This suggests an important association requiring strategies for prevention, management, and mitigation.

### Limitations

There are several important limitations to our study, inherent in the post hoc formation of patient cohorts from a prospective binational, pediatric-intensive care registry. We found a significant increase in pediatric DSH admissions to intensive care after the onset of the pandemic in Australia, but this observational study suggests no conclusions on causation. Other societal, cultural, and policy factors may have been confounders. Similarly, potential intermediary changes in online socialization, hypothesized but unproven before the global outbreak,^44^ were not elucidated in our data and cannot be ruled out under pandemic conditions.^45^ Diagnostic coding in the registry is tailored to describing intensive care episodes, containing little detail on preexisting or concomitant psychiatric diagnoses or history of the presenting illness.

Actual patient-specific factors associated with triggering of DSH, details regarding presence or absence of suicidal intent, and complexities of individual circumstances remained unknown, as did socioeconomic and ethnic data. Further research is required in this area. Additionally, coding did not allow us to unequivocally identify or classify self-injury. While the literature broadly supports the assumption of self-harm in the selected diagnostic codes in this age group,^3,27,46^ misclassification of true accidents cannot be excluded.

Our analysis also indicated another potential break in the temporal trend, in October 2019. While our limited data set and a search of scientific literature and gray literature (including reports and government documents) did not identify any specific factor in late 2019 to explain a prepandemic increase in DSH incidence, it is possible that as-yet unknown factors were associated with the DSH ICU admission rate prior to the global COVID-19 pandemic. This possibility suggests that the onset of the pandemic may be 1 of several factors in the complex genesis of DSH, many of which could not be identified in this analysis.

As an ICU registry study, our analysis did not include patients with DSH who did not present to hospitals, who were admitted to hospitals but did not require intensive care, or who died prior to arrival in ICUs. Also not included were children and adolescents admitted to adult ICUs not contributing to the pediatric registry. A significant number of older adolescents may therefore have been missed, potentially skewing the data, particularly in the group aged 16 to 17 years. This also leaves open the possibility of bias if admission to adult ICUs was patterned by the characteristics of DSH. We therefore cannot rule out the possibility that our data set underestimates the true DSH ICU admission rate for this age group, nor that there was a shift in admissions of older adolescent patients away from adult and toward pediatric ICUs during the pandemic. The latter outcome, however, is unlikely given that there was no difference in patient ages before vs after the break point in the timeline. Overall, with the ANZPIC registry representing more than 90% of pediatric ICU admissions across Australia, we believe our findings are robust.

Additionally, it is important to note that the results cannot necessarily be generalized to other patient cohorts, time periods, or jurisdictions. In particular, our study did not provide a comparison with jurisdictions that were subject to less restrictive public health measures but instead experienced pressures from, for example, a substantially higher burden of SARS-CoV-2–related disease and high excess population mortality.

## Conclusions

In this Australian national registry–based cohort study, the coronavirus pandemic in Australia was associated with a significant increase in admissions of children and adolescents to intensive care with DSH. Addressing this challenge in the pediatric population during the current health crisis may require perspective, policy, and resourcing beyond direct COVID-19 morbidity and mortality.

## Notes

Supplement 1. **eMethods 1.** Interrupted Time-Series Analysis **eMethods 2.** Mixed Effects Regression Model **eResults.** Results for the Model for Primary Outcome

Supplement 2. **Nonauthor Collaborators.** ANZICS PSG and ANZICS CORE
