# Supplementary material for: Admissions of Children and Adolescents With Deliberate Self-harm to Intensive Care During the SARS-CoV-2 Outbreak in Australia
Source: JAMA Netw Open. 2022 May 11;5(5):e2211692. doi: 10.1001/jamanetworkopen.2022.11692 (PMC9096595; doi:10.1001/jamanetworkopen.2022.11692)
Supplement: Supplement 1. — eMethods 1. Interrupted Time-Series Analysis eMethods 2. Mixed Effects Regression Model eResults. Results for the Model for Primary Outcome [file jamanetwopen-e2211692-s001.pdf]

Supplemental Online Content

Corrigan C, Duke G, Millar J, et al; Australian and New Zealand Intensive Care Society Pediatric Study Group (ANZICS PSG); ANZICS Center for Outcome and Resource Evaluation (ANZICS CORE). Admissions of children and adolescents with deliberate self-harm to intensive care during the SARS-CoV-2 outbreak in Australia. *JAMA Netw Open.* 2022;5(5):e2211692. doi:10.1001/jamanetworkopen.2022.11692

eMethods 1. Interrupted Time-Series Analysis
eMethods 2. Mixed Effects Regression Model
eResults. Results for the Model for Primary Outcome

This supplemental material has been provided by the authors to give readers additional information about their work.

© 2022 Corrigan C et al. *JAMA Network Open.*

eMethods 1. Interrupted Time-Series Analysis

Two estimators were used, itsa and xtbreak.

The form of the itsa estimator was:

itsa dsh sex age population pim3, single trperiod(month) lag(n)

where itsa = logical operator; dsh = monthly frequency of deliberate self harm admissions, sex = gender of patient limited to binary male/female; age = patient age in years; population = sex- and age-matched national population; trperiod = date (month) of interruption; lag = maximum of n lags in the autocorrelation structure.

Newey-West standard errors are applied to the estimated coefficients.

The itsa command estimates the effect of an intervention when the outcome variable is ordered as a time series and a number of observations are available in both preintervention and postintervention periods. The model is referred to as an interrupted time-series analysis because the intervention is expected to interrupt the level or trend subsequent to its introduction.

Table 1. Output for itsa and interruption event time set to March 2020.

Covariate	Coefficient	t	P>|t|	95% confidence interval	
time since start of study	0.135	4.67	<0.001	0.078	0.193
COVID-19 (post-March 2020)	2.868	2.31	0.024	0.392	5.344
Interaction term (time.COVID-19)	0.474	3.04	0.003	0.163	0.785
Sex (male=1)	1.241	7.82	<0.001	0.924	1.558
Age(years)	0.453	0.7	0.483	-0.828	1.733
Per 1,000 population	-0.815	-4.37	<0.001	-1.187	-0.443
Severity of illness (PIM3)	2.933	3.03	0.003	1.004	4.861

See manuscript for graphical presentation of these results.

The form of the xtbreak estimator for unknown breaks was:

xtbreak test dsh L1.ym, breakpoints(date , fmt(tm)) vce(hac)

where xtbreak test = logical operator; dsh = monthly frequency of deliberate self harm admissions; L = maximum of n lags in the autocorrelation structure; ym = data grouped by month and year; breakpoints = interruption or intervention date(s); fmt(tm) = date format ; vce = covariance matrix estimator; hac = heteroskedastic and autocorrelation robust

xtbreak test implements multiple tests for structural breaks in time series and panel data models. The number and period of occurrence of the structural breaks may be known or unknown.

Table 2: xtbreak output for proposed interruptions.

Break Point Date	October-2019	March-2020	Both dates
W(tau)	372.05	255.02	212.64
p-value	<0.001	<0.001	<0.001

Interruptions dates set at
October 2019 the statistically identified as possible ('unknown') break point.
March 2020 as the clinically proposed break point associated with COVID-19 onset in Australia.

© 2022 Corrigan C et al. *JAMA Network Open.*

eMethods 2. Mixed Effects Regression Model

Standard models treat time as a (categorical or continuous, as specified) covariate without adjustment for autoregression. They are not preferred for time series analysis. They were, however, included and reported in our analysis for three reasons.

First, the interrupted time series analyses (see Appendix 1) did not identify any significant (unknown) interruptions for the majority of the secondary outcomes. We were therefore concerned to avoid Type II error and miss a clinically significant result. Secondly, our time series analyses were based on (monthly) grouped data and volatility in monthly rates may obscure the influence of important individual patient characteristics. Finally, the study population was a clinically diverse and complex patient group. Mixed effect regression permits analysis of individual patient characteristics.

The form of the estimator was:

melogit dsh i.month population age sex intervention ||state:, vce(robust)

where melogit = mixed effects regression logical operator; dsh = binary (presence/absence) of deliberate self harm admission diagnosis; month = month and year as categorical covariate; sex = gender of patient limited to binary male/female; age = patient age in years; population = sex- and age-matched national population; intervention = dummy variable indicating before or after pandemic onset (March 2020); state = state of origin of hospital admission as a random intercept; vce(robust) = robust errors in covariance matrix estimator.
Akaike (AIC) and Bayesian (BIC) information criteria were used to test model fit.

Results for the model for primary outcome (DSH admission to PICU)

Table 1 Model Covariates with addition of intervention variable.

Model covariate	Odds ratio	z	P-value	95% confidence interval	
Interruption1	4.835	2.07	0.039	1.086	21.528
Month	1.010	4.07	0.000	1.005	1.015
Population2	0.630	-3.44	0.001	0.484	0.820
Sex (male)	0.557	-4.15	0.000	0.422	0.734
Age (year)	1.148	3.70	0.000	1.067	1.235
Month	Data omitted for simplicity				

Interruption = clinically proposed break point associated with COVID-19 onset in Australia; before (0) or after (1) March 2020.
per 10,000 population

Table 2. Information Criteria for Primary Outcome Models

Model	df	AIC	BIC
Without intervention covariate	5	5478.925	5515.253
With intervention covariate	6	5471.409	5515.002

© 2022 Corrigan C et al. *JAMA Network Open.*

eResults. Results for the Model for Primary Outcome

Model Covariates with addition of intervention variable.
Primary outcome was DSH admission to pediatric ICU.

Model covariate	Odds ratio	z	P-value	95% confidence interval	
Intervention[1]	4.835	2.07	0.039	1.086	21.528
Month	1.010	4.07	0.000	1.005	1.015
Population[2]	0.630	-3.44	0.001	0.484	0.820
Sex (male)	0.557	-4.15	0.000	0.422	0.734
Age (year)	1.148	3.70	0.000	1.067	1.235
Month	Data omitted for simplicity				

1. Intervention = before (0) or after (1) March 2020.
2. per 10,000 population

Information Criteria for Primary Outcome Models

Model	df	AIC	BIC
Without intervention covariate	5	5478.925	5515.253
With intervention covariate	6	5471.409	5515.002

© 2022 Corrigan C et al. *JAMA Network Open.*
