# Supplementary material for: Admissions of Children and Adolescents With Deliberate Self-harm to Intensive Care During the SARS-CoV-2 Outbreak in Australia
Source: JAMA Netw Open. 2022 May 11;5(5):e2211692. doi: 10.1001/jamanetworkopen.2022.11692 (PMC9096595; doi:10.1001/jamanetworkopen.2022.11692)
Supplement: Supplement 2. — Nonauthor Collaborators. ANZICS PSG and ANZICS CORE [file jamanetwopen-e2211692-s002.pdf]

*Group Name(s): Australian and New Zealand Intensive Care Society Pediatric Study Group (ANZICS PSG) and the ANZICS Center for Outcome and Resource Evaluation (ANZICS CORE)							
***First Name and Middle Initial(s)**	***Last Name**	***Suffix (eg, Jr, III)**	**Academic Degrees**	**Institution**	**Location (city, state/province, country)**	**Role or Contribution, eg, chair, principal investigator**	**Group (if more than 1 Group listed in the byline) and/or Subgroup (eg, Steering Committee)**
Anusha	Ganeshalingam		MBBS FCICM	Starship Children's Hospital	Auckland, New Zealand	Review and approval of study design, Approval of manuscript	Australian and New Zealand Intensive Care Society Pediatric Study Group (ANZICS PSG)
Claire	Sherring		RN	Starship Children's Hospital	Auckland, New Zealand	Review and approval of study design, Approval of manuscript	Australian and New Zealand Intensive Care Society Pediatric Study Group (ANZICS PSG)
Simon	Erickson		MD FCICM	Perth Children's Hospital	Perth, Western Australia, Australia	Review and approval of study design, Approval of manuscript	Australian and New Zealand Intensive Care Society Pediatric Study Group (ANZICS PSG)
Samantha	Barr		RN	Perth Children's Hospital	Perth, Western Australia, Australia	Review and approval of study design, Approval of manuscript	Australian and New Zealand Intensive Care Society Pediatric Study Group (ANZICS PSG)
Sainath	Raman		MBBS MRCPCH PhD FCICM	Queensland Children's Hospital and the University of Queensland	Brisbane, Queensland, Australia	Review and approval of study design, Approval of manuscript	Australian and New Zealand Intensive Care Society Pediatric Study Group (ANZICS PSG)
Debbie	Long		RN	Queensland Children's Hospital and the University of Queensland	Brisbane, Queensland, Australia	Review and approval of study design, Approval of manuscript	Australian and New Zealand Intensive Care Society Pediatric Study Group (ANZICS PSG)
Luregn	Schlapbach		MD PhD FCICM	University Children's Hospital Zurich	Zurich, Switzerland	Review and approval of study design, Approval of manuscript	Australian and New Zealand Intensive Care Society Pediatric Study Group (ANZICS PSG)

*First Name and Middle Initial(s)	*Last Name	*Suffix (eg, Jr, III)	Academic Degrees	Institution	Location (city, state/province, country)	Role or Contribution, eg, chair, principal investigator	Group (if more than 1 Group listed in the byline) and/or Subgroup (eg, Steering Committee)
Kristen	Gibbons		PhD	Child Health Research Centre, The University of Queensland	Brisbane, Queensland, Australia	Review and approval of study design, Approval of manuscript	Australian and New Zealand Intensive Care Society Pediatric Study Group (ANZICS PSG)
Shane	George		BSc MBBS FACEM MPH	Gold Coast University Hospital	Gold Coast, Queensland, Australia	Review and approval of study design, Approval of manuscript	Australian and New Zealand Intensive Care Society Pediatric Study Group (ANZICS PSG)
Puneet	Singh		MBBS MD FCICM	Sydney Children's Hospital	Sydney, New South Wales, Austrlia	Review and approval of study design, Approval of manuscript	Australian and New Zealand Intensive Care Society Pediatric Study Group (ANZICS PSG)
Vicky	Smith		RN	Sydney Children's Hospital	Sydney, New South Wales, Australia	Review and approval of study design, Approval of manuscript	Australian and New Zealand Intensive Care Society Pediatric Study Group (ANZICS PSG)
Carmel	Delzoppo		BappSc	The Royal Children's Hospital	Melbourne, Victoria, Australia	Review and approval of study design, Approval of manuscript	Australian and New Zealand Intensive Care Society Pediatric Study Group (ANZICS PSG)
Ben	Gelbart		MBBS FRACP FCICM	The Royal Children's Hospital	Melbourne, Victoria, Australia	Review and approval of study design, Approval of manuscript	Australian and New Zealand Intensive Care Society Pediatric Study Group (ANZICS PSG)
Subodh	Ganu		MD MEpi	Women’s and Children’s Hospital	Adelaide, South Australia, Australia	Review and approval of study design, Approval of manuscript	Australian and New Zealand Intensive Care Society Pediatric Study Group (ANZICS PSG)

*First Name and Middle Initial(s)	*Last Name	*Suffix (eg, Jr, III)	Academic Degrees	Institution	Location (city, state/province, country)	Role or Contribution, eg, chair, principal investigator	Group (if more than 1 Group listed in the byline) and/or Subgroup (eg, Steering Committee)
Georgia	Letton		RN	Women's and Children's Hospital	Adelaide, South Australia, Australia	Review and approval of study design, Approval of manuscript	Australian and New Zealand Intensive Care Society Pediatric Study Group (ANZICS PSG)
Marino	Festa		MD FCICM	The Children's Hospital at Westmead	Sydney, New South Wales, Australia	Review and approval of study design, Approval of manuscript	Australian and New Zealand Intensive Care Society Pediatric Study Group (ANZICS PSG)
Gail	Harper		RN	The Children's Hospital at Westmead	Sydney, New South Wales, Australia	Review and approval of study design, Approval of manuscript	Australian and New Zealand Intensive Care Society Pediatric Study Group (ANZICS PSG)
Jan	Alexander		n/a	Child Health Research Centre, The University of Queensland	Brisbane, Queensland, Australia	Provision of national dataset	ANZICS Center for Outcome and Resource Evaluation (ANZICS CORE)
Breanna	Pellegrini		Bcomm	Australian and New Zealand Intensive Care Society (ANZICS)	Melbourne, Victoria, Australia	Provision of national dataset	ANZICS Center for Outcome and Resource Evaluation (ANZICS CORE)
